# Arc discharge rapid synthesis of engineered copper oxides nano shapes with potent antibacterial activity against multi-drug resistant bacteria

**DOI:** 10.1038/s41598-022-24514-w

**Published:** 2022-11-23

**Authors:** Bassma H. Elwakil, M. Toderas, Mostafa El-Khatib

**Affiliations:** 1 Department of Medical Laboratory Technology, Faculty of Applied Health Sciences Technology, Pharos University in Alexandria, Alexandria, 21526 Egypt; 2 Department of Physics, University of Oradea, 410087 Oradea, Romania; 3 Basic Sciences Department, Faculty of Engineering, Pharos University in Alexandria, Alexandria, 21526 Egypt

**Keywords:** Microbiology, Diseases, Medical research, Materials science, Nanoscience and technology

## Abstract

Nowadays Nano metals have received an eminent compromise of attention. Even though different nanostructure of same metal maybe gives different results in wide range applications. Copper oxide (CuO-NPs) and Copper Nano wires (CuO-NWs) were prepared in controlled size via the alternating current Arc discharge process. Deionized water and argon gas were the chosen dielectric medium during the process to obtain 2 different forms of copper oxides. By changing the dielectric material from deionized water to argon gas the shape of CuO nanoparticles changed from spherical (CuO-NPs) to wires (CuO-NWS). The yield prepared depicted the purity of the prepared CuO, and their diameters were about 10 ± 5 nm and 30 ± 3 nm for CuO-NWs and CuO-NPs respectively. In vitro cytotoxic effect of the prepared CuO-NWs & CuO-NPs using human normal lung fibroblast cell line (WI-38 cells) revealed that CuO-NWs & CuO-NPs CC_50_ values were 458.8 and 155.6 µg/mL respectively. Both yields showed potent antibacterial activity against different multi-drug resistant *Acinetobacter baumannii* strains. A complete eradication of the bacterial growth was noticed after 4 Hrs incubation with CuO-NWs. Moreover, CuO-NWs showed superior antibacterial activity (with minimum inhibitory concentration reached 1.8 µg/mL) over CuO-NPs. The detailed antibacterial activity mechanism of CuO-NWs was further investigated; data proved the precipitation and adsorption of the nanoparticles on the bacterial cell surface leading to cell deformation with reactive oxygen species increment. The results explicated that the nanoparticles shape plays an essential role in the antibacterial activity. Rotational Arc discharge machine might be a promising tool to obtain various metal nanostructures with low cost and environmentally friendly with potent activity.

## Introduction

Bacterial resistance to antibiotics has increased dramatically over the past two decades, and it presents a serious worry for medical experts worldwide. An efficient way to inhibit bacteria is to use metal nanoparticles due to their tiny size that immobilizes the activity of bacteria^1^. Saravanan et al.^2^ reported the emerged drug resistance mechanism through extended spectrum β-lactamases enzyme which can hydrolyze a variety of β-lactam antibiotics. In this contest, Multidrug resistant bacteria (MDR) have emerged as a serious problem for public health which resulted from antibiotics abuse and misuse^3^. One of the most prevalent MDR bacteria is *Acinetobacter baumannii*. *A. baumannii* belongs to Gram-negative bacilli (GNB) causing extensive drug resistance (XDR) and carbapenem resistant (CRAb) infections which limit its treatment options^4^. Many hospitals acquire infections caused by *A. baumannii* (pneumonia, septicemia and blood stream infections (BSI)) resulting in a significantly high mortality rate among hospitalized patients^5^. Colistin (COL) was the last resort for XDR and CRAb infections, though the resistance to polymyxins (including colistin) had been widely and rapidly reported^6^. This raised a struggle to discover new active compounds against colistin resistant strains^7^. Nanotechnology has been a promising field in the combat against the ever-growing number of antimicrobial-resistant microorganisms, especially through metal nanoparticles application^8,9^. Nanoparticles have different characteristics compared to bulk materials of identical chemical composition. Since the mechanistic action of nanoparticles is to attack many sites and targets within the bacterial cell, no microbial resistance was reported against metal nanoparticles^10^.

Nano-metals have piqued the scientific imagination due to their excellent electrical, optical, and structural properties^11–13^. Metals oxides have a variety of attractive characteristics, making it suitable for use in a wide range of applications^12^. Semiconductor transition metal oxide Nano-architecture as construction blocks has received important attention due to their novel structures and characteristic impact on multi-discipline applications recently. Cu nanoparticles showed great potential applications in medicine^14^, agriculture^15^, devices and fabrications^16^. Many research labs have been used to manufacture copper nanostructure with different shapes by various methods. More precisely, crystal structure could be considered as one of the important effective factors on the properties and applications of NPs. Synthesis of Nano-metal oxides often involves toxic compounds especially when using surface capping and reducing agent via chemical methods^17^. The disadvantage of preparing nano-metal oxides in chemical methods vanished by using Arc discharge method in the presence of deionized water which is an alternative, not expensive, effective and environmentally friendly method avoiding toxicity that may cause cell damage^18^. In general, Arc discharge routines usually used to create metal nanoparticles with spherical shape under normal conditions. The unique properties of Copper oxide (CuO-NPs) and Copper Nano wires (CuO-NWs) piqued the interest of researchers, who have devoted special attention to Nano research and applications^14–17^. The properties of the produced Nano material were remarkably influenced by the technique of production^11^. Indeed, Arc discharge method successfully produced Nano-metals from the metal electrodes^12^. The parameters variance such as temperature^14,19^, pressure^19,20^, electrode shape^12^, the gap between electrodes^21^, current^14^, the voltage applied^22^, type of power supply^22^, and dielectric medium have great effect on the nanoparticle’s size and shape yields^18,23^. On the other hand, numerous studies showed that copper oxides have a great effect on bacterial inhibition against *Escherichia coli*, and a mild inhibitory effect on other bacterial strains^9^. Moreover, the antimicrobial activity of CuO-NPs has also been reported against *Pseudomonas aeruginosa, Escherichia coli, Klebsiella pneumoniae, Staphylococcus aureus, Bacillus subtilis*, and methicillin-resistance *Staphylococcus aureus* (MRSA)^9^. Recently, few reports studied the bactericidal activity of CuO-NPs which was found to generate reactive oxygen species (ROS) causing bacterial cell death^24^. The CuO-NPs size greatly affects the antibacterial activity (the smaller nanoparticles size, the higher antibacterial activity)^24^. CuO-NPs directly bind to the bacterial cell wall, interact with the membranes’ proteins that lead to membrane perforation and releasing intracellular materials^25^. Some researches mentioned that Cu ions may interact with the sulfur and phosphorus-containing biomolecules (DNA and proteins) that lead to structure distortion^26,27^.

Thus, the aim of this study was to produce CuO-NPs and CuO-NWs with low toxicity by both solid–liquid or solid–gas Arc discharge methods due to their high biological activity against bacteria. The study also aimed to examine the yield morphology produced using a high resolution transmission electron microscope (HR-TEM), to investigate the nanoparticles crystallinity using the X-ray diffraction analyzer (XRD), inductively coupled plasma optical emission spectrometry (ICP-OES) to know the exact amount produced, and the sample stability was measured through Zeta potential. On the top of that, the growing urge to find new therapy in the combat against colistin resistant strains obligate us to explore the potential antibacterial activity of CuO-NPs and CuO-NWs against some colistin-resistant *Acinetobacter baumannii* strains. Hence, this is the first work to emphasize the antibacterial activity of Copper in different shapes against colistin-resistant bacterial strains.

## Materials and methods

**Figure 1:**
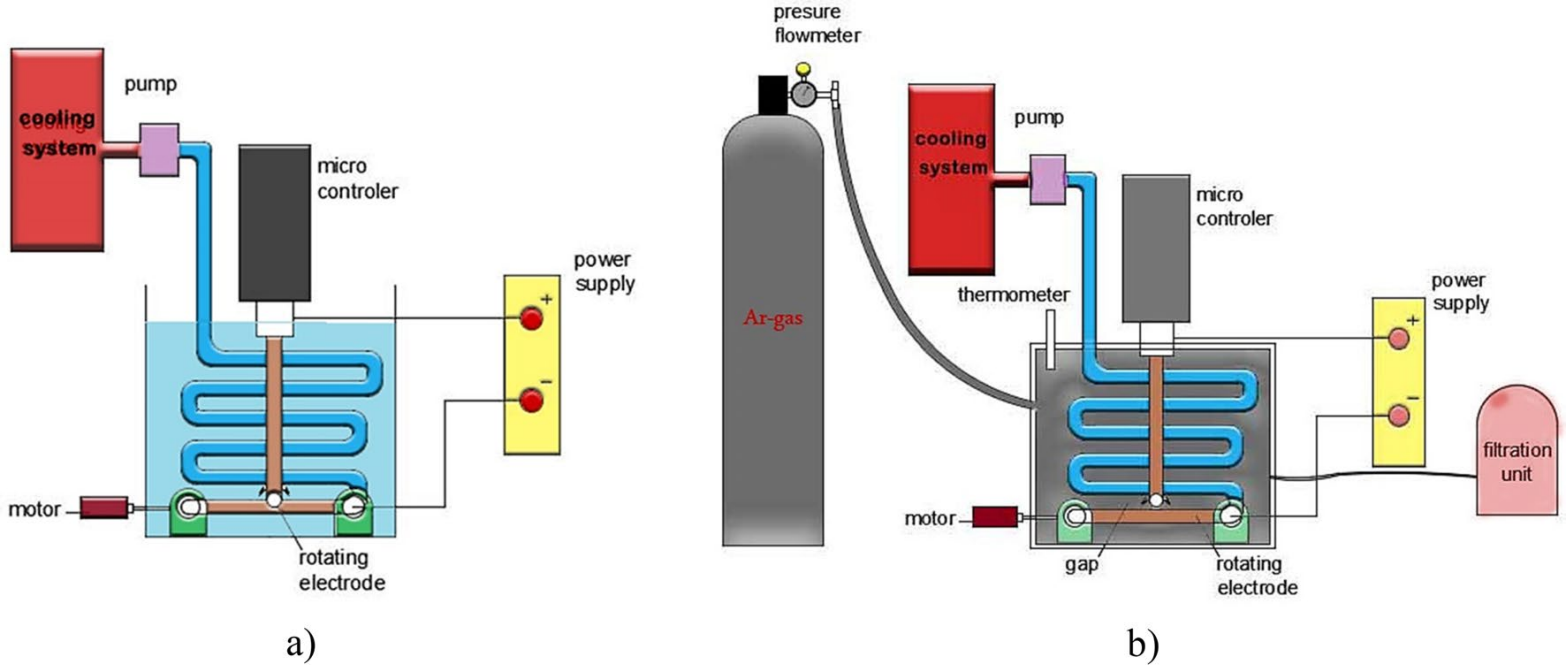
Arc-discharge machine unit using (**a**) deionized water (**b**) Ar gas.

High purity CuO-NPs and CuO-NWs were synthesized by the Arc discharge method under fixed conditions of electrode dimensions, alternating current, power supply, voltage used, vessel capacity, rotational speed, pH, electrode gap, and discharge duration as tabulated in Table 1. A voltage of 70 V with reasonable current 15 A is used to maintain a steady Arc discharge that would improves the yield quality and quantity in both experiments. Another key component is the cathode's rotational speed at 950 rpm that accelerates metal clustering and prevents it from condensation on cathode surface. This parameter has a significant impact on the particle size properties and stability. The cathode is taken in large dimension with respect to the anode to increase the yield. The experiment was carried twice by changing dielectric material. Copper electrodes dispersed in deionized water (conductivity = 0.8–0.9 µS) were used to produce CuO-NPs at temperature about 20 °C as shown in Fig. 1a, then copper electrodes were used to get CuO-NWs using argon (Ar) gas as dielectric media with a temperature of 100 °C as shown in Fig. 1b. The process was free of chemical agents. The transition of metal vapors into Nano yield may be divided into three stages: nucleation, cluster growth, and condensation in deionized water^2^.

**Table 1:** The important parameters used in the system.

Key parameters	Values	
	System A	System B
Discharging voltage (average value)	70 V	70 V
Discharging AC—current (average values)	15A	15A
Cathode disk (diameter, length or thickness)	16 mm,10 cm	16 mm,10 cm
Anode (diameter, length)	2 mm,3 cm	2 mm,3 cm
Discharging duration time	30 min	30 min
Temperature of solution (before& after)	20 °C	100 °C
Pressure	103 Kpa	160 Kpa

### Filtration and characterization

Filtration was carried out by using a cooling ultracentrifuge model (Hettich MIKRO, German) separation of the nanoparticles was according to their mass where the lower layer contains massive particle. The relative centrifugal force (RCF) was then calculated by applying Eq. (1) ^21^.

1 $$ {\text{RCF}} = 1.118{ } \times 10^{ - 5} \times {\text{r}} \times {\text{N}}^{2} $$

where RCF is the relative centrifugal force (cm/sec^2^), r is the rotational radius (cm), and N is the rotating speed (revolution per minute, rpm). Then each sample was characterized by JEOL JEM-2100 HR-TEM, XRD, and Zetasizer (nano 90 Malvern, UK).

### In vitro studies

#### Evaluation of cytotoxic effects of the prepared nanoparticles

Human lung fibroblast (WI-38 cells) normal cell line (ATCC® number: CCL-75™) was obtained from the American Type Culture Collection (ATCC, Rockville, MD). MTT assay is a quantitative method that can be used to measure the cells’ viability. Precultured cell line (5 × 10^4^ cell/well in Corning® 96-well tissue culture plate) was exposed to eight serially diluted concentrations, incubated for 24 Hrs then the numbers of viable cells were determined by the MTT test. The relation between surviving cells and drug concentration was plotted to get the survival curve of WI-38 cell line after treatment with CuO-NPs and CuO-NWs one at a time according to Eq. (2). The cytotoxic concentration (CC_50_), the concentration required to cause toxic effects in 50% of intact cells, was estimated from graphical plots of the dose response curve for each tested concentration using Origin Pro6.8 software^28^.

2 $$ {\text{Cell Viability\% }} = {\text{OD of treated cells}}/{\text{OD of untreated cells }} \times 100{\text{ \% }} $$

#### Antibacterial activity

##### Disc diffusion method

The antibacterial activities of CuO-NPs and CuO-NWs were evaluated against different *Acinetobacter baumannii* colistin resistant strains. 1.5 × 10^6^ CFU/ml (0.5 McFarland) bacterial suspensions were prepared, 100 μl of each was swabbed over the surface of Müeller-Hinton agar (MHA) plate. Disc-diffusion method was carried out to assess the antibacterial activity; each sterilized disc (Whatman No. 1 with 6 mm diameter) was saturated with 25 μl of 20 mg CuO-NPs and CuO-NWs individually then placed over the inoculated MHA plates. Incubation for 18 Hrs at 37 ± 2 °C was done^29,30^. Colistin resistant strains of *Acinetobacter baumannii* under test were kindly identified and provided by the Microbiology Department’s Strain Bank, et al.-Shatby Pediatric Hospital, Alexandria, Egypt.

##### Minimum inhibitory concentration (MIC)

MIC test was done by adding a mixture of 20 μl tween 80, 80 μl of sterile Müeller-Hinton broth and 100 μl of CuO-NPs and CuO-NWs individually. The mixture was serially diluted using a two-fold dilution in 96-well microtiter plate. 100 μl of 0.5 McFarland bacterial suspensions was inoculated in each well. MIC is known as the minimum inhibitory concentration of the tested drug to inhibit the bacterial growth^29,30^.

##### Bacterial lethality curve

Time-kill curve was investigated to estimate the optimum time required for CuO-NPs and CuO-NWs to kill the bacterial vegetative cells. CuO-NPs and CuO-NWs were added individually to 1 ml Müeller-Hinton broth by using the nanoparticles MIC values then 1 ml of the bacterial inoculum was added. Aliquots from each tube were taken to assess the bacterial growth through different incubation time (0, 2, 4, 6, 8, 12 and 24 Hrs) at OD 600 nm^29^.

##### Transmission electron microscope study

Transmission electron microscopic (TEM) examination (JEM-100 CX, JOEL, Japan), has a resolution of 3 nm at 30 kV. Buffers and dehydration protocol were used by graded acetone series and epoxy resin embedding. Ultrathin sections were prepared on grids then stained with 3% uranyl acetate^29,31^.

##### Reactive oxygen species content measurement

Reactive oxygen species (ROS) was measured by a HPF (3′-(phydroxyphenyl fluorescein) where 5 mM HPF was added to 200 μL bacterial suspension treated with 100 μg/mL of the most potent nanoparticles. The plate was incubated in dark at 37 °C, and fluorescence of each well was measured at 0, 1, 2, and 3 h, respectively, with excitation/emission at 490/515 nm^32^.

## Results and discussions

### Preparation and characterization of the Nano yields

**Figure 2:**
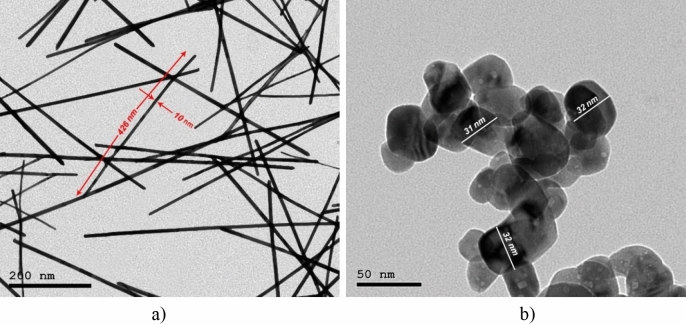
HR-TEM of CuO-NWs (**a**) and CuO-NPs (**b**).

Uniform cuprous oxides with different morphologies have been successfully synthesized using different pressure and temperature during Arc discharge process. After solid–liquid phase Arc discharge process for CuO-NPs preparation, the vessel contained suspended nanoparticles dispersed in deionized water. To extract the nanoparticles from the solution, the radius and rotational speed of the ultracentrifuge were adjusted to 7.5 cm and 10,000 rpm for 30 min, respectively. HR-TEM study was performed for each sample (Fig. 2b). The results showed that CuO-NPs have spherical shape with average diameter 41 ± 5 nm.

**Figure 3:**
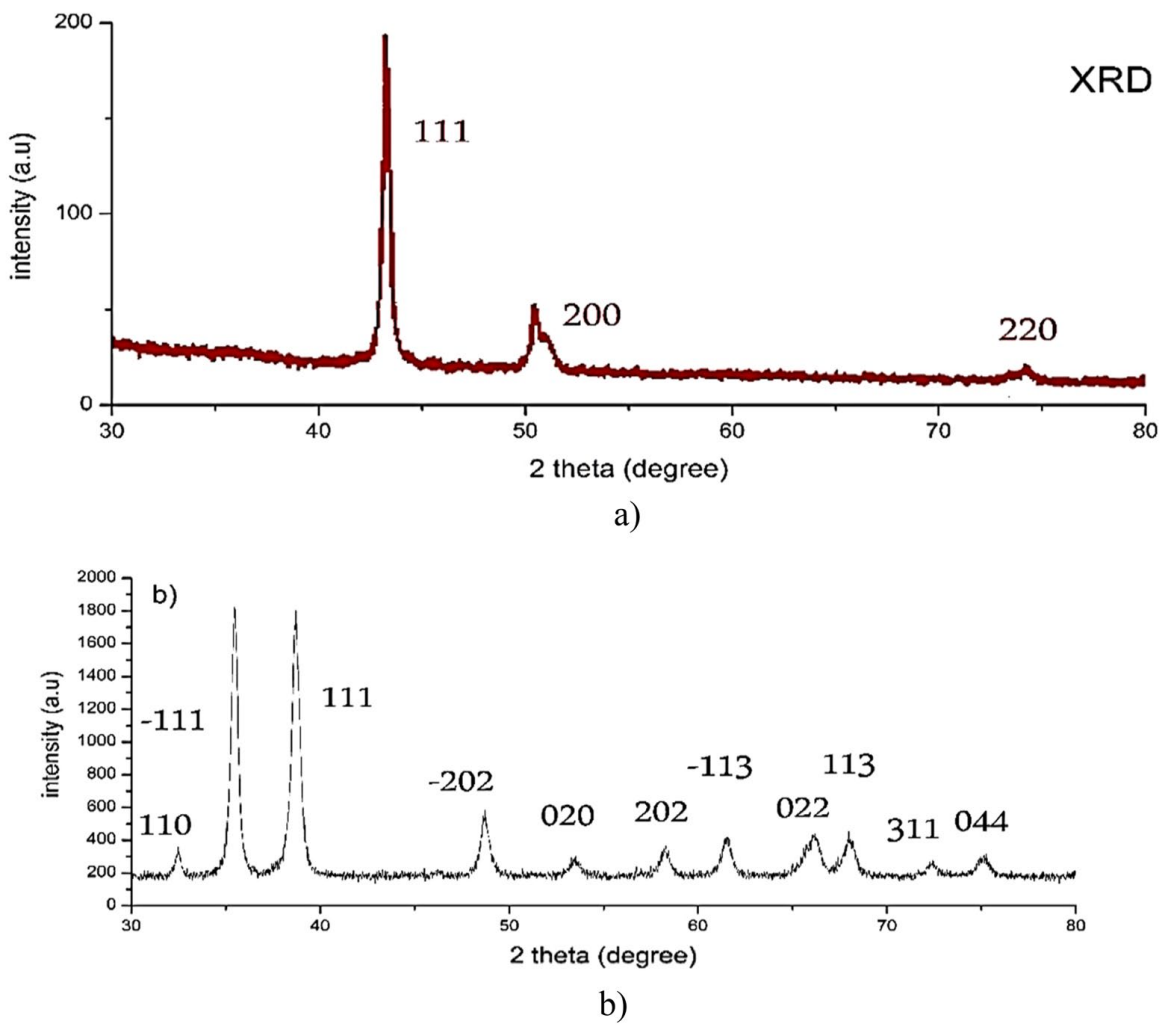
XRD pattern of (**a**) CuO-NWs and (**b**) CuO-NPs.

On the other hand, HR-TEM depicts CuO-NWs (Fig. 2a) as a thin wire shape in the Nano range with diameter 9 ± 3 nm and length from 3 to 5 µm which were easily collected after 10 min from switching off the Arc discharge unit. It’s clear that the surface area of CuO-NWs was greater than CuO-NPs. CuO-NPs and CuO-NWs were prepared in different conditions which resulted in different shapes. Figure 3a illustrated the XRD pattern of each sample. The pattern assigned to intensities of three major peaks revealed the presence of CuO-NWs. The high intense peak for face centered cubic (fcc) structure was in good agreement with (JCPDS #03-1018). On the other hand, XRD (Fig. 3b) revealed sharp diffraction peaks at 2θ indexed for highly pure CuO-NPs using JCPDS file number 01-080-0076. The XRD data and HR-TEM results confirmed that the prepared samples were pure (without impurities). Both CuO-NWs and CuO-NPs were crystalline materials. When nanoparticles were immersed in deionized water, they created an area of electrical inhomogeneity at the solid–solution interface.

As a result, an apparent excess of charge at the solid surface was precisely balanced by a diffuse area of another opposite charge. This diffused area, which originated in the aquatic environment, was known as the electrical double layer. This process may cause nanoparticles’ aggregation; therefore, it is important to measure the electrical potential of the interface between the aqueous solution and the stationary layer which is known as zeta potential. Zeta potential was measured for CuO-NWs & CuO-NPs (−47 ± 1.4 and −34 ± 1.8 mV, respectively). The high zeta potential confirmed the stability of the prepared samples.

**Figure 4:**
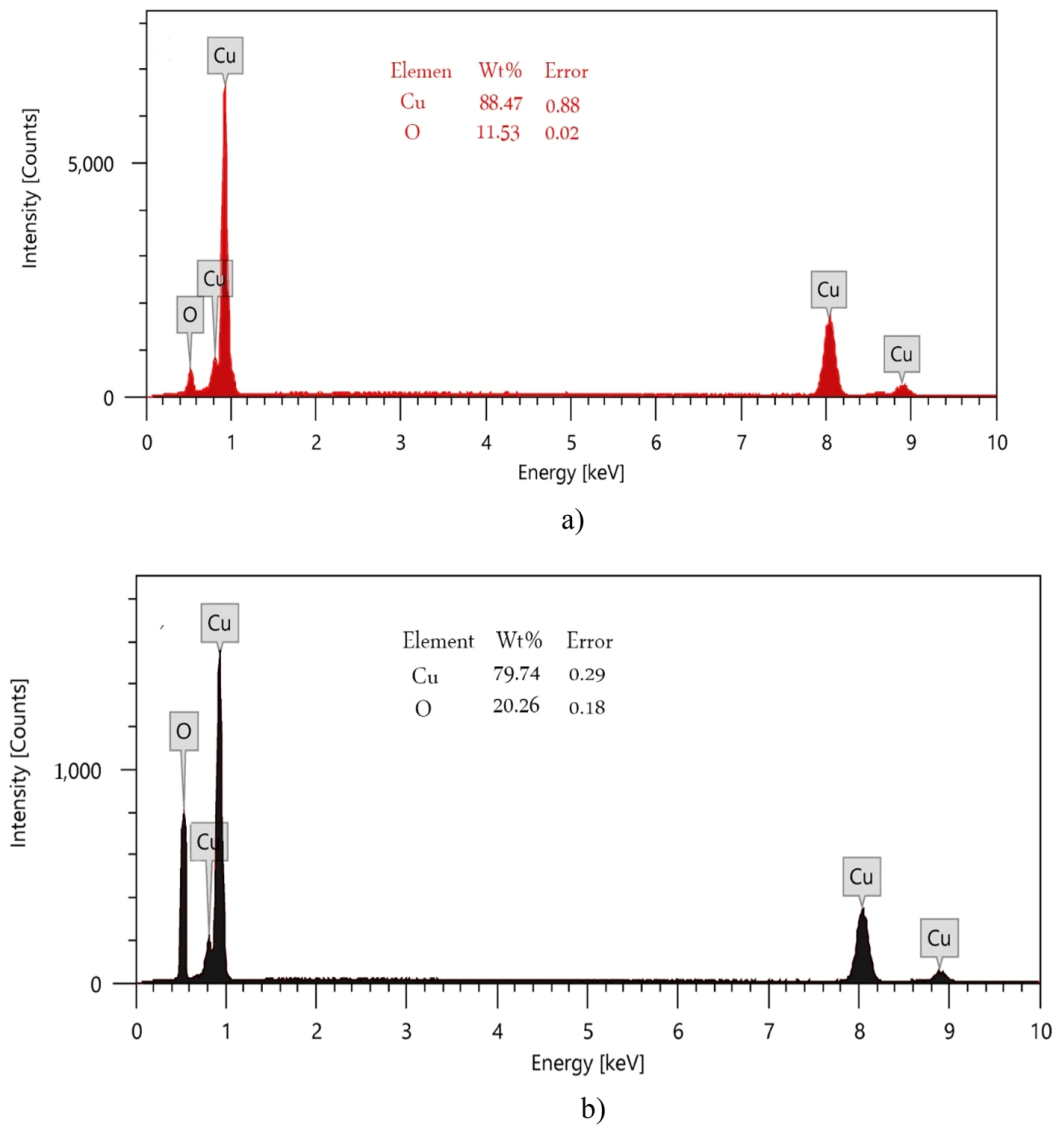
EDX analysis of (**a**) CuO-NWs and (**b**) CuO-NPs.

EDX analysis CuO-NWs results were displayed in Fig. 4-a, which declared that the prepared nanoparticles were almost pure Cu_2_O. On the other hand Fig. 4-b showed a uniform distribution of copper to oxygen with atomic ratio of 1:1 in CuO-NPs which had higher presence of Oxygen atoms compared to CuO-NWs. This result ensured the formation of pure nano-yields.

ICP-OES Device was used to examine exactly the amount of produced Nano copper yields prepared with different temperature and pressure. According to these measurements the masses of the prepared samples namely CuO-NWs or CuO-NPs were found to be 72 ± 2 mg/min and 54 ± 1 mg/min respectively. According to the abovementioned investigations, to successfully make the gliding Arc between two electrode gaps, strongly apply field with sufficient energy must done to eliminate the electron from an electrode (and behind metal ions) in gas or liquid. It was found that the Arc discharge preparation method for the two Nano-systems resulted in different yield shape which may be related to the change of pressure and temperature that affect the work function which was responsible for eliminating the electron forming metal ions nanoparticles.

### Cytotoxic effects of the prepared nanoparticles

**Figure 5:**
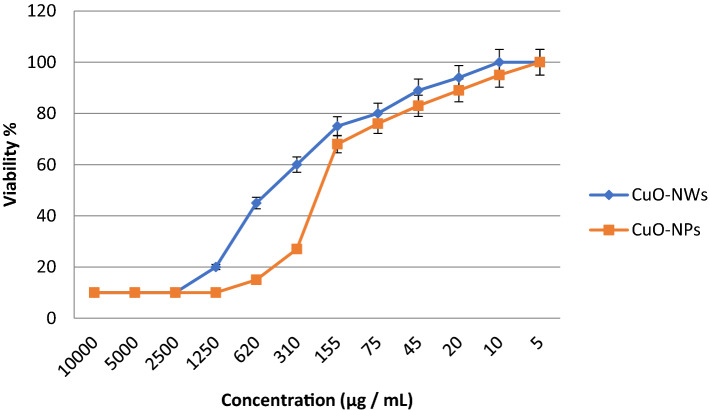
The percentage of normal fibroblast cells viability upon treatment with CuO-NWs & CuO-NPs one at a time.

In a trial to study the in vitro cytotoxic effect of the prepared CuO-NWs & CuO-NPs the cell proliferation using human normal lung fibroblast cell line (WI-38 cells) was tested. Lung fibroblast cell line was chosen due to the possible biomedical application of the prepared CuO nanoparticles as a potent treatment against *Acinetobacter baumannii* infection. It was found that at 620 µg/mL of CuO-NWs, and CuO-NPs, the fibroblast cell viability were 45 and 15% respectively. CuO-NWs & CuO-NPs CC_50_ values were 458.8 and 155.6 µg/mL respectively (Fig. 5). Fahmy et al.^33^ studied the Cu/CuO-NPs effect on normal lung cells and reported IC_50_ value reached 201.26 µg/mL.

### Antibacterial activity

**Figure 6:**
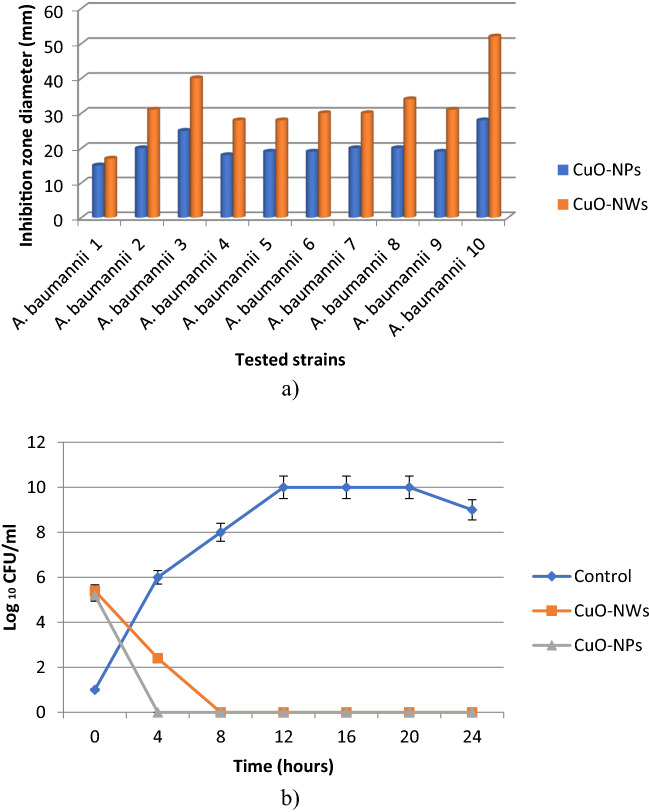
Antibacterial effect of CuO-NWs & CuO-NPs against *Acinetobacter baumannii* strains represented in inhibition zone diameter (**a**), and the bacterial lethality curve (**b**).

**Figure 7:**
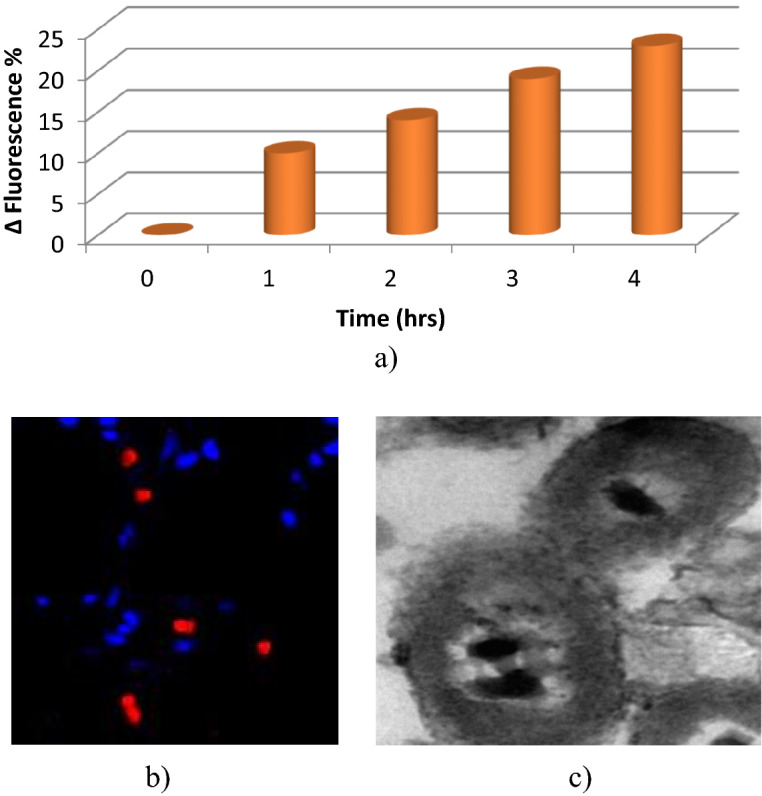
Antibacterial effect of CuO-NWs & CuO-NPs against *Acinetobacter baumannii* strains represented in ROS generation with a fluorescent HPF probe (**a**), fluorescent images of *Acinetobacter baumannii* cells after treatment with live/dead fluorescent dyes to indicate cell membrane permeability (**b**) and transmission electron microscopic (TEM) study (**c**).

Antibacterial activity of CuO-NWs & CuO-NPs were evaluated against Colistin resistant strains of *Acinetobacter baumannii* through various techniques namely disc diffusion method, minimum inhibitory concentration (MIC), bacterial lethality curve, HR-TEM study, ROS content measurement and NADH content measurement. Data revealed that CuO-NWs had higher antibacterial activity than the nanoparticles spherical form. This may be explained by the smaller size with larger surface area of the prepared nanowires compared to the nanoparticles form. *A. baumannii* strain number 10 was the most susceptible strain while *A. baumannii* strain number 1 was the most resistant one (Table 2, Fig. 6a). The bacterial lethality curve was assessed against the most resistant strain (*A. baumannii* strain number 1). It was noticed that CuO-NWs completely eradicated the bacterial growth after 4 h contact time while CuO-NPs inhibited the bacterial growth after 8 h (Fig. 6b). This test confirmed the superior antibacterial activity of CuO-NWs. The antibacterial mechanism of CuO-NWs was studied through different techniques against the most resistant strain (*A. baumannii* strain number 1). It has been previously verified that metal nanoparticles usually inhibit the bacterial growth through disrupting the bacterial cell membrane and metal ions releasing that enters the bacterial cytoplasm and disrupt the protein functions. This assumption was confirmed through the HR-TEM study of the treated bacterial cells which revealed the CuO precipitation and adsorption on the bacterial cell surface leading to cell deformation (Fig. 7c). The bacterial cell response to such stimuli has usually resulted in a reactive oxygen species (ROS) increment. ROS content measurement was assessed with a commercial fluorescent probe, it was observed that the ROS content increased by 23% after 4 h incubation with the potent CuO-NWs (Fig. 7a). Moreover, the observed red fluorescent dead cells were observed which confirmed the CuO antibacterial mechanism (cytoplasmic membranes inactivation and bacterial cells apoptosis due to cellular membrane disruption) (Fig. 7b).

**Table 2:** Antibacterial effect of CuO-NWs & CuO-NPs against *Acinetobacter baumannii* strains.

Tested strains	CuO-NPs		CuO-NWs	
	IZ (mm)	MIC (µg/mL)	IZ (mm)	MIC (µg/mL)
*A. baumannii* 1	15.0	250.0	17.0	250.0
*A. baumannii* 2	20.0	125.0	31.0	30.0
*A. baumannii* 3	25.0	30.0	40.0	3.7
*A. baumannii* 4	18.0	250.0	28.0	30.0
*A. baumannii* 5	19.0	250.0	28.0	30.0
*A. baumannii* 6	19.0	250.0	30.0	30.0
*A. baumannii* 7	20.0	125.0	30.0	30.0
*A. baumannii* 8	20.0	125.0	34.0	15.0
*A. baumannii* 9	19.0	250.0	31.0	15.0
*A. baumannii* 10	28.0	30.0	52.0	1.8

Lv et al.^34^ stated that Cu-NPs inhibited the bacterial growth of *Staphylococcus aureus* and *Escherichia coli* isolates by cytoplasmic membranes inactivation. The Cu-induction of bacterial ROS leads to stimulation of DNA strand cleavage^34^. Chatterjee et al.^35^ revealed that Cu-NPs can inhibit the bacterial growth via various mechanisms namely lipid peroxidation, ROS generation, protein oxidation and DNA degradation in *E. coli* cells. Other studies mentioned that the antibacterial mechanism of action of Cu-NPs was due to the reactive complex formation between the cellular medium organics and CuO-NPs^36^. Gunawan et al.^37^ also confirmed the antibacterial mechanism of CuO-NPs due to copper-peptide complex formation that lead to multiple fold increase of the bacterial ROS resulted in the overall inhibition of biomass growth. Kumar et al.^38^ reported that the CuO nanostructures with high surface area showed higher antimicrobial activity.

The mechanism of action of cupric (Cu^2+^) and cuprous (Cu^1+^) ions in inhibiting the bacterial growth was discussed by Midander et al.^39^ who stated that the Cu^2+^ ions liberation from CuO nanostructures could disrupt the bacterial cell membrane and finally deactivate the microbial growth. Jadhav et al.^40^ proposed the antibacterial activity of CuO-NPs can be attributed to the induction of ROS originated from the electron–donating nature of copper oxides. Meghana et al.^41^ mentioned that the antibacterial activity of CuO and Cu_2_O nanoparticles is highly and closely related to the metal oxidation states which follow the independent pathway of the cell membrane disruption and bactericidal/bacteriostatic activity. It was also reported that the ROS pathway can be applied to CuO nanoparticles than Cu_2_O. The antibacterial effect of cupper oxides against *E.coli* was proposed as that the Cu_2_O usually form copper(I)–peptide complex while CuO cause free radical generation. CuO nanoparticles produced significant ROS in terms of super oxides while Cu_2_O did not. Another study investigated the antibacterial activity of CuNPs against multispecies biofilm, and it was proved that CuNPs have an immediate action with gradual increasing through time reaching their highest antibiofilm effect after 7 days incubation^42^. Moreover, a time-kill curve kinetics of Cu-NPs declared that the complete microbial growth eradication can be observed within 4 Hrs incubation^43^.

## Conclusion

In summary, CuO-NWs with diameter 9 ± 3 nm and length from 3 to 5 µm showed higher stability compared with spherical shape of CuO-NPs with average diameter 41 ± 5 nm . Different copper oxide shapescan be selectively synthesized by the choice of a suitable pressure and temperature. The difference between energy applied and the work function to eliminate electrons may be transferred to heat energy in dielectric media and/or play a vital role in reshaping the crystal order of the formed ions to produce specific shape. In vitro cytotoxic effect of the prepared CuO-NWs & CuO-NPs using human normal lung fibroblast cell line (WI-38 cells) revealed that CuO-NWs & CuO-NPs CC_50_ values were 458.8 and 155.6 µg/mL respectively. The antibacterial activity of CuO-NWs was higher than CuO-NPs and that could be explained by that the cupric (Cu^2+^) and cuprous (Cu^1+^) ions inhibited the bacterial growth by disrupting the bacterial cell membrane and ROS induction. According to the high antibacterial activity and relatively low toxicity, Cu-NWs could be used as a new potent treatment against colistin and multi-drug resistant infections.

## Data Availability

The datasets used and/or analyzed during the current study available from the corresponding author on reasonable request.
